# Relative Contributions of Packaging Elements to the Thermal Hysteresis of a MEMS Pressure Sensor

**DOI:** 10.3390/s20061727

**Published:** 2020-03-19

**Authors:** Youssef Hamid, David A. Hutt, David C. Whalley, Russell Craddock

**Affiliations:** 1 Wolfson School of Mechanical, Electrical and Manufacturing Engineering, Loughborough University, Loughborough, Leicestershire LE11 3TU, UK; 2 Druck Ltd. a division of Baker Hughes, 2 Fir Tree Lane, Groby, Leicestershire LE6 0FH, UK

**Keywords:** packaging levels, consecutive packaging, piezoresistive pressure sensor, thermal hysteresis, polynomial fitting

## Abstract

Piezoresistive silicon pressure sensor samples were thermally cycled after being consecutively packaged to three different levels. These started with the absolute minimum to allow measurement of the output and with each subsequent level incorporating additional packaging elements within the build. Fitting the data to a mathematical function was necessary both to correct for any testing uncertainties within the pressure and temperature controllers, and to enable the identification and quantification of any hysteresis. Without being subjected to any previous thermal preconditioning, the sensors were characterized over three different temperature ranges and for multiple cycles, in order to determine the relative contributions of each packaging level toward thermal hysteresis. After reaching a stabilised hysteretic behaviour, 88.5% of the thermal hysteresis was determined to be related to the bond pads and wire bonds, which is likely to be due to the large thermal mismatch between the silicon and bond pad metallisation. The fluid-fill and isolation membrane contributed just 7.2% of the total hysteresis and the remaining 4.3% was related to the adhesive used for attachment of the sensing element to the housing. This novel sequential packaging evaluation methodology is independent of sensor design and is useful in identifying those packaging elements contributing the most to hysteresis.

## 1. Introduction

Piezoresistive single crystal silicon (Si) based sensors are widely used to measure pressure in numerous applications, including aerospace, oil and gas, and industrial [1,2,3,4]. Their transduction mechanism is based upon the external pressure causing the deflection of a thinned section of Si that forms a diaphragm. This deflection is sensed through the use of piezoresistors embedded in the Si surface which act as strain gauges connected in a Wheatstone bridge configuration, thereby transforming the applied pressure into a voltage output. This mechanism has been widely studied over the last 50 years, where the mechanical properties of single crystal Si [5,6,7] and its piezoresistive properties at different doping concentrations [8,9,10,11,12] have been well established. Enhanced models, which account for both the anisotropy of Si and thermal effects on the piezoresistivity, have also been recently published [13,14,15,16,17,18,19]. This provides an excellent basis for initial sensor design, but cannot be solely relied upon to accurately predict the output of a complete pressure sensor. This is because the isolated sensing element (SE), which the theoretical models describe, cannot function as a practical pressure sensor if it is not mechanically supported and protected, and its electrical output made accessible to electronic instrumentation, i.e., it is packaged. Therefore, various packaging elements are required to transform the SE into a functioning sensor, but these may themselves affect the diaphragm deflection and/or the measured voltage output of the SE. While considering the SE alone was acceptable in the past, researchers and designers are recognizing the need to account for the effects of packaging elements as significant performance drivers for complete piezoresistive pressure sensors [20,21], particularly where devices are required to operate with higher precision and stability over longer operational lifetimes and within more demanding operating environments.

Pressure sensors can have various mechanical elements supporting and protecting the SE, such as adhesives [22], frits [23], glob tops [24], isolation fluids [25], and metal membrane caps [26]. Electrical elements connecting the piezoresistors to signal conditioning electronics can include the bond pad metallisations on the SE surface [27] along with, for example, wire bonds [28], solders [29,30], or more complex flip chip connections [31]. The role of each of these elements on the performance of a SE is not fully understood and their impacts will vary with the particular overall sensor design. Developing a universal transduction model capable of predicting the effect of such a wide range of packaging elements on the SE output would therefore be challenging; however, if the most significant contributors of a particular design can be identified then this will facilitate further improvements. With this in mind, the primary aim of this study is to propose a novel methodology, independent of sensor design, for assessing the relative effects of packaging elements on the performance of a chosen device. This would enable the quantification of the individual contributions and highlight the areas for focus in any future design optimisation.

Piezoresistive pressure sensors have been reported to exhibit thermal dependency similar to that reported by Liu et al. [19]. Thermal dependence can have its source either in the Si itself or in the elements used to package the SE. The stiffness of Si has both first and second order temperature coefficients [32], while there is also a thermal component to its piezoresistive coefficient, as reported by Kanda [12]. Si also has a temperature coefficient of resistance that is a function of doping levels. During device manufacturing, and throughout its operational lifetime, temperature-dependent package-related stresses will inevitably be transferred to the SE, due to different thermal expansion coefficients and other temperature dependent behaviour of the package. The SEs are usually designed so that any symmetrical stresses affecting the piezoresistors within the Wheatstone bridge are theoretically cancelled out. However, due to manufacturing tolerances and the assembly sequences, packaging will rarely impose perfectly balanced stresses that remain constant with time. The combined effects of most of these dependencies can be eliminated if the temperature of the SE is known and a mathematical fitting function [21,31] is applied to the raw voltage output to correct for any thermal contributions to the pressure signal. When the effects of these stresses cannot be compensated away, their effects will be falsely misinterpreted as pressure inputs that the sensor is designed to measure and will therefore negatively impact the performance of the sensor.

As will be described later, when thermally cycled, the stresses imposed on the SE by the packaging elements may lead to device hysteresis, i.e., the output voltage for the same applied pressure will vary depending on the thermal history [33]. However, to accurately quantify any hysteresis requires the sensor output to be recorded at identical pressure and temperature values during both the increasing and decreasing phases of the temperature cycle. In practice, due to limitations of the measurement system as well as in the pressure and temperature controllers, the applied pressure and temperature always vary slightly from the desired set values. An interpolation of the raw data to a fitted value at exactly the required point is consequently necessary to account for these variations and to ensure the calculated hysteresis is due to packaging effects and not due to errors in the test and measurement system. Therefore, a secondary aim of this paper is to establish the benefit and necessity of fitting the data as a means of accurate identification of the presence of sensor hysteresis and its quantification.

There have been a number of published studies on thermal hysteresis of piezoresistive pressure sensors [21,31,34,35]. All investigated fully packaged sensors, and to the authors’ knowledge, none have investigated the relative contributions of the packaging elements involved in a particular design to the overall hysteresis. Waber et al. [31] characterised a flip-chipped absolute pressure sensor cleverly decoupled from its substrate using flexible copper springs. The hysteresis of the sensor was studied over a −30 to 70 °C temperature range at 100 kPa by applying a fitting function to the sensor’s raw output. The hysteresis was reduced from 140 to 20 Pa by reducing the width of the metallisation on the active face and increasing the depth of the reference cavity. Unfortunately, neither details of the number of pressure and temperature cycles used to obtain the fitting function, nor its mathematical description, were presented. Sandvand et al. [21] used a linear regression applied to the third thermal cycle to measure the hysteresis effect due to a 0.8 μm-thick Aluminium (Al) metallisation layer deposited directly on top of the piezoresistors in an absolute SE designed for pressures up to 100 kPa. These SEs were glass frit bonded to a Si stress isolation layer, which itself was glass frit bonded to a TO-8 header. The study reported that the units underwent ten –55 to 125 °C thermal cycles followed by a stabilisation bake for 24 hours at 150 °C. The sensors having the extra Al metallisation on top of the piezoresistors exhibited an additional error of approximately 250 Pa (0.25% of full scale) compared with those without the extra Al. The thermal hysteresis figures in these papers were determined over different temperature ranges and the sensors did not undergo the same thermal preconditioning. It is, therefore, unfortunately not possible to directly compare their results and draw any further conclusions on the effects of the packaging on thermal hysteresis.

In summary, two specific aims were addressed in this study. First, a packaging and testing methodology was derived in order to identify the relative contributions of packaging elements to thermal hysteresis over several temperature cycling ranges. Without being subjected to any thermal pre-treatment, the same SEs were packaged in consecutive configurations, so that differences due to variability from SE to SE could be eliminated. As a hypothesis, it is postulated that packaging effects are cumulative in nature and that one could predict the overall hysteresis by knowing the effect of each group of packaging elements. If this is correct, then the methodology of decomposing the entire package into “sub-packages” could be generalized to other packaging configurations incorporating different sets of packaging elements. The second aim of this work was to establish the requirement for fitting the sensor’s output voltage as a means of eliminating any test and measurement inaccuracies to reveal and quantify hysteresis as a function of the thermal cycling regime. This included an investigation of the effect of increasing orders of polynomial fitting function, leading to the use of the same optimal polynomial order to compare hysteresis values between the different packaging levels.

## 2. Packaging and Thermal Effects

### 2.1. Sensing Element Packaging Levels

The SEs investigated in this study were experimental units that were selected due to their relatively low operating pressure range (50 to 200 kPa), which makes them more sensitive to any packaging effects. To describe the stages of the packaging process and the packaging elements involved in the realisation of the pressure sensor, the micro-electromechanical systems (MEMS) “packaging levels” terminology as proposed by Krondorfer and Kim [20] was used, whereby increasing levels of packaging imply an increasing number of elements incorporated within the sensor build. Table 1 shows this system of packaging levels, as applied to the sensor investigated here, as well as the packaging elements potentially affecting the SE for each level. The L0 package comprised of a 1.65 mm × 1.65 mm × 0.37 mm single crystal Si SE with a 22 μm thick etched diaphragm. Defined regions were doped to create the four piezoresistors on the top face, with one located on each side of the 810 μm × 810 μm square diaphragm, and were connected in a Wheatstone bridge configuration. A diode junction was also fabricated within the structure of the SE, which was used to monitor its temperature for subsequent fitting. Seven 1 μm thick Al–Si bond pads were sputtered on the top face of the SE at 150 °C and sintered at a temperature between 400 and 450 °C, before returning to ambient temperature. The SEs were then anodically bonded at a temperature between 350 and 400 °C, under vacuum, to a 1 mm thick layer of borosilicate glass, creating a sealed reference pressure, P_ref_, beneath the diaphragm, before again returning to ambient temperature. These L0 packages were then singulated from the wafer and prepared for the subsequent packaging steps. It should be noted that the L0 package cannot be considered as a pressure sensor, as the SE and borosilicate glass are not mechanically supported. Furthermore, the electrical output cannot be measured due to the absence of connections to external instrumentation. Hence, this packaging level was not tested and is denoted as zero, or L0.

In the first packaging level, L1, the L0 packaged SE was mechanically supported and electrically connected to operate as a pressure sensor. The L0 package was attached to a stainless steel housing using a standard two-part epoxy structural adhesive, while 38 μm diameter Al–Si wire bonds were used to electrically connect the SE bond pads to the feedthrough pins of the housing. For the L1 package a user would be able to apply pressure and measure the electrical output, and therefore it can be considered as a pressure sensor exposed to the pressure media.

For harsh environments, where the SE needs to be isolated from the pressure media, additional packaging elements are required [4,36,37]. In the L2 package, a 25 μm thick stainless steel isolation diaphragm was electron-beam welded to the housing in order to completely seal out the external environment from the SE. The enclosed cavity was also filled under vacuum with an inert dielectric fluid to ensure no air was trapped. Within the operating pressure range, the fluid was considered to be incompressible and to convey pressure from outside the L2 package to the SE without pressure loss. The L2 packaged sensor was the highest packaging level considered in this study.

### 2.2. Pressure Response and Thermal Effects

When the Wheatstone bridge is supplied with a constant voltage, *V_s_*, it is customary to divide the Wheatstone bridge voltage, *V_b_*, by *V_s_* to obtain the normalised output voltage, referred to hereafter as the voltage output, *V_out_*. This enables comparison with other sensors that are not supplied with the same voltage.

(1) $$V_{out}=\frac{V_{b}}{V_{s}}\;\;$$

**Figure 1:**
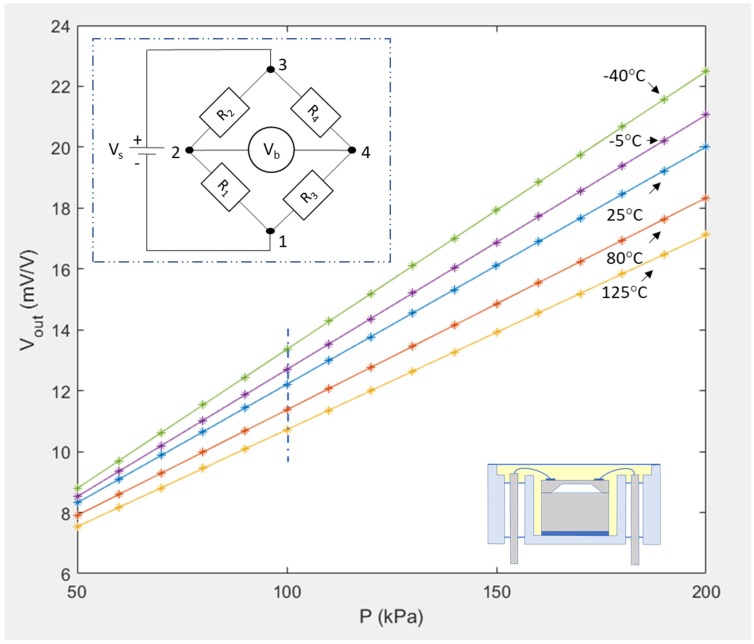
Voltage output response to a 50 to 200 kPa pressure input at different temperatures for one of the L2 packaged sensors used in this study. The inset shows the Wheatstone Bridge configuration and associated voltages (R_1_, R_2_, R_3_, R_4_ represent the piezoresistors).

It should be noted that *V_s_* is not the voltage provided by the external power supply to the pressure sensor, but the voltage supplied to the bridge, which can be different. *V_out_* is proportional to the applied pressure, *P*, for a given temperature, *T*, as shown in Figure 1. Although pressure sensors can suffer from a non-linear response due to applied pressure [38], for the small pressure ranges considered here, this will not affect the particular design described in this paper. Due to the thermal dependence, at 100 kPa, denoted by the vertical dashed blue line in Figure 1, the sensor output ranges from 10.7 mV/V at 125 °C to 13.4 mV/V at −40 °C. This represents a 20% variation, which can be largely compensated by applying a fitting function.

**Figure 2:**
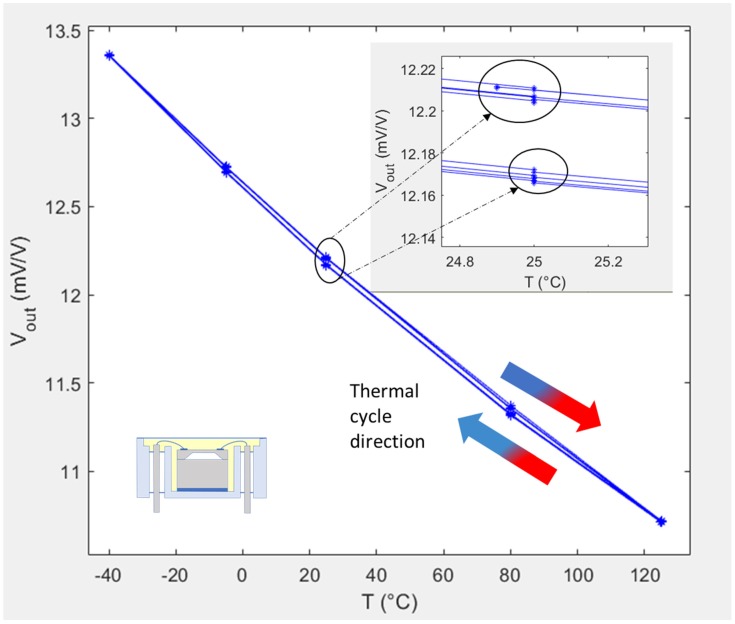
Voltage output response at 100 kPa for one of the L2 packaged sensors used in this study and subjected to multiple thermal cycles from −40 to 125 °C.

When cycling the temperature of the sensor multiple times and recording its output for the same applied pressure, thermal cycling effects start to appear. As can be seen in Figure 2, particularly in the enlarged inset, at around 25 °C the output depends on whether the sensor has been cooled to 25 °C from 125 °C or heated to 25 °C from −40 °C, and the responses for each cycle do not lie exactly on top of each other. This thermal history dependency of the output is a thermal hysteresis phenomenon [33].

For the sensor showcased in Figure 2, the hysteresis, *H,* at 25 °C and 100 kPa during the first cycle can be calculated as the difference between the output voltage, V_out_ ↗, at 25 °C, when approached from cold at 100 kPa, and the output voltage, V_out_ ↘, at 25 °C, when approached from hot at 100 kPa. The hysteresis was therefore calculated as 45.5 μV/V based on:

(2) $$H\left(25{}^{\circ}C,\;100kPa\;\right)=\left[V_{out}↗\left(25{}^{\circ}C,100kPa\;\right)-V_{out}↘\left(25{}^{\circ}C,100kPa\;\right)\right]$$

This hysteresis value would be correct if one were to be sure that the data points *V_out_*↗*,* and *V_out_*↘, were recorded at exactly 100 kPa and 25 °C. In reality, the applied pressure and temperature always vary slightly from the desired set values, due to limitations of the pressure and temperature controllers. However, as the actual values of *T* (obtained from *V_d_)* and *P* at the point of data collection are recorded, an interpolation of the raw data to a fitted value at exactly 100 kPa and 25 °C can be applied to account for these variations.

## 3. Testing and Data Fitting

### 3.1. Testing Sequence

The same SEs were packaged in three consecutive levels of packaging. In addition to the L1 and L2 packaging configurations, as described in Table 1, a floating L1 configuration (L1-F) was also achieved as an intermediate packaging step between the standard L0 and L1 configurations. Without being subjected to any prior thermal preconditioning, ten units were initially packaged in this L1-F configuration, following the method described by Hamid et al. [39]. The sensing elements in this configuration were supported by only the wire bonds and were therefore considered to be “floating”. The 10 units then went through the full sequence of temperature cycling tests before being repackaged in the L1 configuration. The repackaging consisted of carefully removing the existing wire bonds, adhesively bonding the same SEs to the housing using a standard two-part epoxy structural adhesive, and finally wire bonding the die to the feedthroughs for a second time. Only five of the original SE units were successfully repackaged in this way, due to failure of some of the wire bonds. These remaining five SEs were then tested following the same thermal and pressure cycling regime before being further processed to the L2 package configuration, as described previously, and again finally tested.

**Figure 3:**
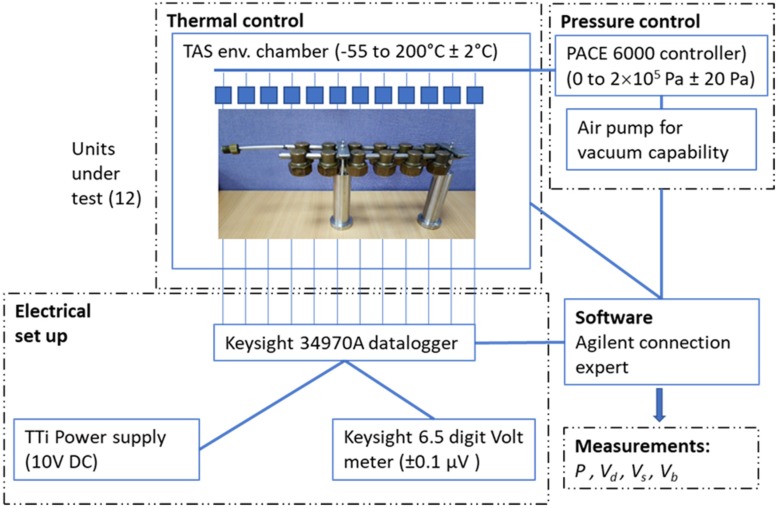
Pressure and temperature cycling test configuration.

The system used to temperature and pressure cycle test the different packages was identical to the one previously described by the authors [39]. The sensors were mounted to a metal manifold, as shown in Figure 3, and a Druck “PACE6000” was used to control the air pressure. The manifold and SEs were placed in a TAS environmental chamber with electrical connectors and cables to convey the voltages to be measured using a Keysight 6.5 digit resolution digital voltmeter and an external datalogger. As discussed in [39], the electrical instrumentation and pressure controller were not inside the environmental chamber and were therefore not subjected to any significant thermal cycling, but only to small fluctuations of the test laboratory ambient (25 °C ± 2 °C). It was therefore assumed that the thermal variation of the measurement system did not affect the accuracy of the measurements of the sensors under testi. Although an effect of humidity on piezoresistive sensors has been reported [40], humidity of the pressurised air was not monitored nor controlled during these experiments and was assumed not to have any significant impact on thermal hysteresis.

**Figure 4:**
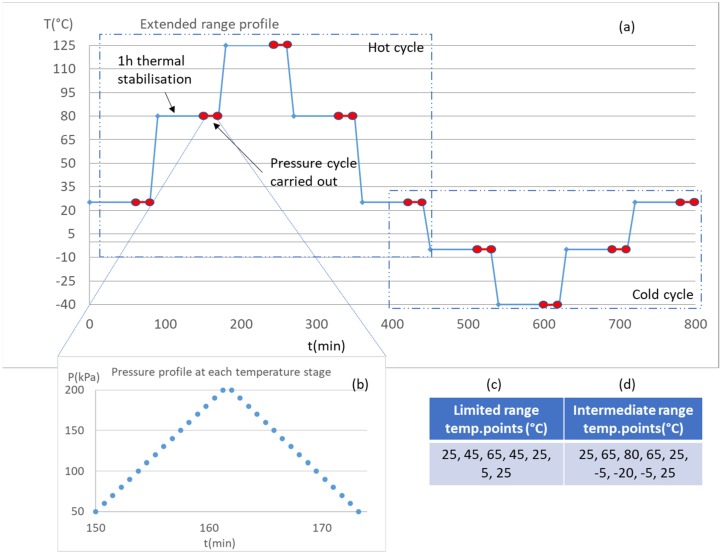
Temperature and pressure cycles applied to the samples: (**a**) the −40 to 125°C extended range temperature profile; (**b**) the 50 to 200 kPa pressure cycle applied at each temperature step within the thermal cycle; and (**c**,**d**) temperature set points for the limited and intermediate thermal cycles.

The units underwent either three or four temperature cycles, starting and ending at 25 °C. The cycles had either “limited” (5 to 65 °C), “intermediate” (−20 to 80 °C), or “extended” (−40 to 125 °C) ranges. One complete thermal cycle for the extended thermal range is shown in Figure 4a, along with the temperature set points for the two other ranges (Figure 4c,d). After reaching each temperature set point, a 60 min thermal stabilisation time was allowed for the sensors to reach thermal equilibrium. For every temperature point of each thermal cycle, the sensors were subjected to a 50–200 kPa pressure cycle, as highlighted in Figure 4b. A 30 s pressure stabilisation time was allowed at each pressure set point to let the controller equalize the pressure within the manifold. The voltages *V_b_, V_s_*, and the diode voltage, *V_d_*, were measured by the datalogger for each pressure set point. In this study, the rates of change of temperature and pressure were neither recorded nor controlled.

### 3.2. Polynomial Fitting and Hysteresis

Table 2 lists the voltage readings (*V_d_*, *V_s_*, *V_b_*) together with the applied manifold pressure (*P*) for an L1-F sample at a nominal pressure of 100 kPa recorded as the pressure was increased from 50 kPa. All of the temperature points for the first limited range cycle are shown. Firstly, it can be seen that, for all temperature points, the pressure readings differ slightly from the 100 kPa set pressure, with a maximum difference of 3.6 Pa (which is within the specification of the pressure controller). Secondly, at the repeated temperatures of 25 °C, the diode voltage deviated by ±219 μV, which represents a temperature variation of ±0.11 °C (using a nominal −1.9 mV/°C diode linear thermal coefficient).

To accurately calculate the sensor’s hysteresis, the pressure and diode voltages, *P* and *V_d_*, must be interpolated to their values at exactly 100 kPa and 25 °C. This could only be achieved using a fitting function, such that any remaining differences in output voltage could then be attributed to hysteresis caused by the temperature cycles and/or packaging, and not to variations in the applied pressure or temperature. Therefore, each studied sensor is characterized by a unique polynomial fitting function *f*(*P*,*V_d_*) that is generated by fitting all the *V_out_* values (Equation (1)), obtained at each of the measured pressure and temperature points for all cycles. The least squares method (minimising the sum of the squared errors) is used to obtain the best fit and leads to a series of fitting coefficients, *a_00_, a_01_, a_10_, …, a_ij_,* depending on the order of polynomial used. The voltage output, *V_out_*, can then be compared to the fitted output voltage, *Vf_out_*, predicted from the measured input parameters, *P* and *V_d_*, which leads to an associated fitting error, *e*. This can be written as:

(3) $$V_{out}=Vf_{out}+e=f\left(P,V_{d}\right)+e$$

The magnitude of the error can be used to assess any non-repeatable behaviour in the sensor’s performance: for a hysteresis free unit the error would be zero, as the fitting function would have eliminated the error of the pressure and temperature sources and compensated for any non-linear, yet-repeatable behaviour of the sensor.

**Figure 5:**
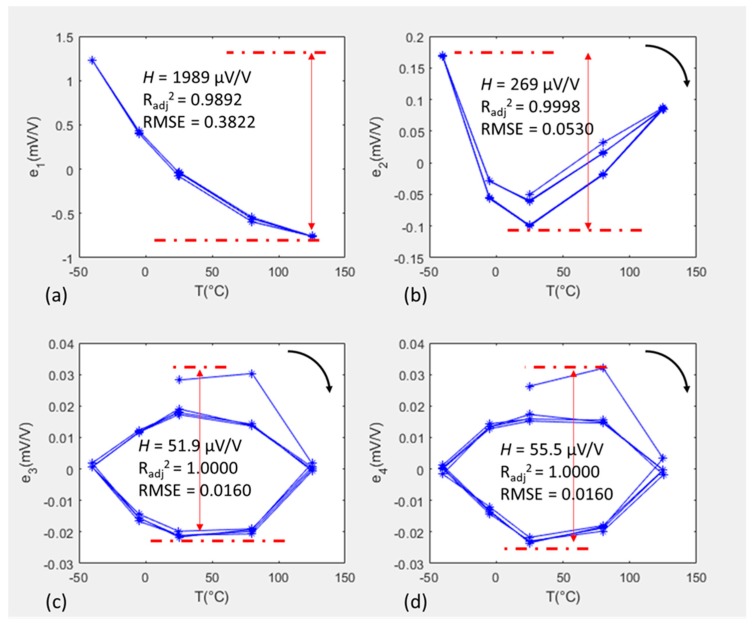
100 kPa data for Unit #9 when L1 packaged and subjected to four extended temperature cycles. The figures show the errors after applying different order polynomial fits: (**a**) 1st order; (**b**) 2nd order; (**c**) 3rd order; and (**d**) 4th order.

Choosing the order of the polynomial fit is important, as higher order fits do not necessarily lead to a reduced error, especially when dealing with hysteretic behaviour that cannot be fitted using polynomial functions. First, second, third, and fourth order polynomial functions were applied to each sensor, and Figure 5 shows the resulting errors for SE #9 packaged in a L1-F configuration and cycled over the extended temperature range. Similar trends were found for all of the other sensors and across the different thermal cycling ranges.

When comparing the error ranges for the successive polynomial orders, it was found that the error evolved from 1989 to 269 to 51.9 to 55.5 μV/V, going from a first to a fourth order. Assessing the evolution of the fitting error in Figure 5, it can be seen that the first and second order fits were able to eliminate the linear and quadratic thermal dependency. Applying the third order fit, the remaining error reveals hysteresis, which a fourth order fit was not capable of eliminating. This was confirmed by the root mean square errors stabilizing after the third order to 0.016, and the adjusted R squared value plateauing at a value of 1. A third order polynomial fit was therefore used, as it was able to eliminate non-linearities and reveal hysteresis, while higher order polynomials provided no discernible improvement. Choosing the lowest polynomial order possible is also, preferable as it simplifies the subsequent electronic signal processing. Therefore, the third order polynomial applied for the voltage output, *Vf_out_*, took the form of:

(4) $${\mathrm{Vf}}_{\mathrm{out}}=a_{00}+a_{01}V_{d}+a_{10}P+a_{02}V_{d}{}^{2}+a_{11}{\mathrm{PV}}_{d}+a_{20}P^{2}+a_{03}V_{d}{}^{3}+a_{12}{\mathrm{PV}}_{d}{}^{2}+a_{21}P^{2}V_{d}+a_{30}P^{3}$$

As the packaged sensors were fitted with a unique set of coefficients for each temperature cycle range, it was assumed that the error, *e*, is determined by physical behaviour which is dominated by hysteresis. In this study, as a worst case scenario, hysteresis, *H*, is defined as the full error range, after a 3rd order polynomial fit, *e*_3_, over all temperature cycles, even though the maximum and minimum error did not occur at the same temperature:

(5) $$H=Max\;\left(e_{3}\right)-Min\;\left(e_{3}\right)$$

For example, in Figure 5c, the maximum error occurred at 80 °C and the minimum occurred at 25 °C, yielding a hysteresis value of 51.9 μV/V. For a typical full-scale output (FSO) of 20 mV/V, this value can be expressed as 0.26%FSO. Unlike the work of Sandvand et al. [21], wherein only the last cycle was used to determine the fitting coefficients, in this study, it was chosen to include all thermal cycles in the calculation of hysteresis. Taking this approach therefore includes the first few data points of the first cycle, which in the third and fourth order plots, can be seen to not follow the trend of the hysteresis in the later cycles. Although the sensor appears to have reached a stable behaviour after four thermal cycles, pausing the test and restarting it may lead to a repetition of the initial behaviour, and therefore this possibility needs to be included within the full sensor performance characterisation process. Such a scenario could occur if the sensor were to be stored for an extended period of time in between cycles.

## 4. Packaging Element Effects on Sensor Hysteresis

In this study, it was decided that the SEs would not be subject to any thermal preconditioning prior to testing and that all fitting functions, although unique to every sensor, would be of the same mathematical form and polynomial order. This would therefore enable a direct comparison between hysteresis errors of differently packaged pressure sensors. Hysteresis can be evaluated at different pressures, but to simplify the study, all hysteresis effects were calculated only at 100 kPa for the five SEs at each successive packaging level from L1-F to L2. These were tested and data-processed for the limited (5 to 65 °C), intermediate (−20 to 80 °C), and extended (−40 to 125 °C) thermal ranges (LR, IR, and ER respectively). The packaging effects on hysteresis are presented for SE #9, but all of the other four repackaged SEs displayed similar trends.

### 4.1. Temperature Cycle Range Effect on Hysteresis

**Figure 6:**
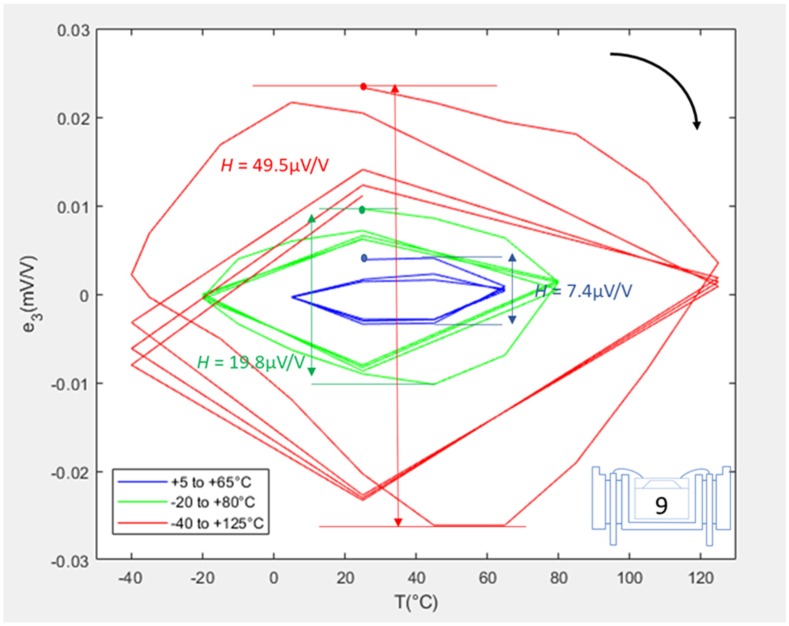
Error plots for SE #9 when L1-F packaged.

Figure 6 shows the fitting error loops for SE #9 when packaged in the L1-F configuration. The blue, green, and red loops represent the LR, IR, and ER respectively. Each loop starts with a filled dot representing the start of the first cycle, and the top right arrow shows the thermal cycling direction. For the LR (blue loop), the test was stopped before the end of the fourth cycle, and therefore hysteresis could only be calculated for the first three cycles.

The first cycles of the IR and ER loops (green and red) looked quite different from their subsequent three cycles. This is due to the fact that, for this packaging configuration only, more intermediate temperature points with respect to the original design of experiment were included in the testing. These were added, as the scale of hysteresis effects was not fully understood at that initial stage of testing. It was, furthermore, chosen to only make measurements at the extreme temperature points and at 25 °C throughout the remaining three cycles to shorten the test time. The hysteresis values were calculated as 7.4, 19.8, and 49.5 μV/V for the LR, IR, and ER respectively, and two observations were made. Firstly, as expected, hysteresis increased with increasing temperature range (a factor of 7 increase between the LR and ER). Secondly, for all temperature ranges, the fitting error loops largely stabilised and became repeatable after just one full temperature cycle.

**Figure 7:**
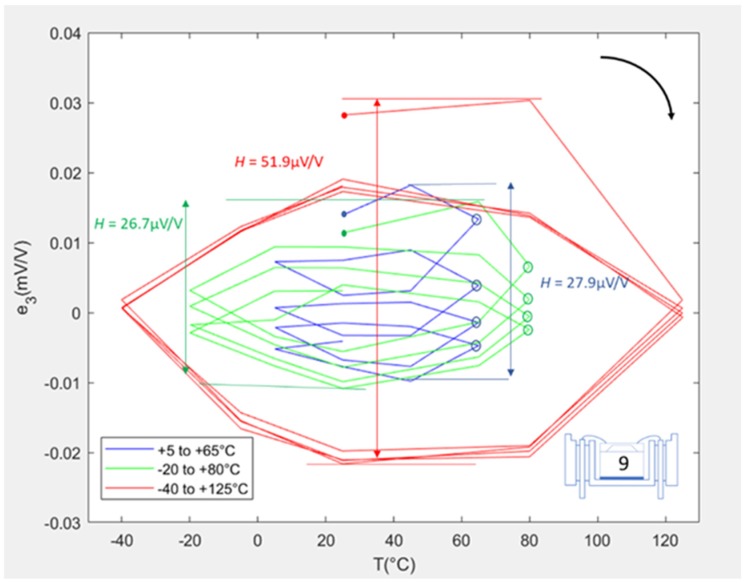
Error plots for SE #9 when L1 packaged.

Figure 7 shows the fitting error loops for the same SE when repackaged in the L1 configuration. The hysteresis values were calculated as being 27.9, 26.7, and 51.9 μV/V for the three cycle ranges. Unlike the L1-F package, the LR and IR loops exhibited a “ratcheting” effect wherein the loop moved downwards with each cycle, although this behaviour was not displayed during the extended temperature range cycles. As the tests were performed consecutively and the ratcheting effect was not present in the L1-F package, the ratcheting behaviour was attributed to the additional presence of the adhesive and housing elements in the L1 package. Of these two packaging elements, it is thought likely that the ratcheting is primarily due to the inelastic nature of the adhesive (a two-part epoxy) rather than the housing (stainless steel). It was also noticeable for both the LR and IR that the ratcheting appeared to be stabilising, i.e., the change in offset with each cycle was reducing. This can be seen by analysing the difference in the consecutive error values at the maximum temperatures of the loops (i.e., the blue and green circles at the 65 and 80 °C points, respectively). In contrast, the loop for the extended temperature range stabilised after only one cycle. In this case, it was observed that the first two points (i.e., at 25 and 80 °C) of the extended loop were significantly outside of the following stabilised loops, which led to the first cycle dominating the hysteresis of the unit.

**Figure 8:**
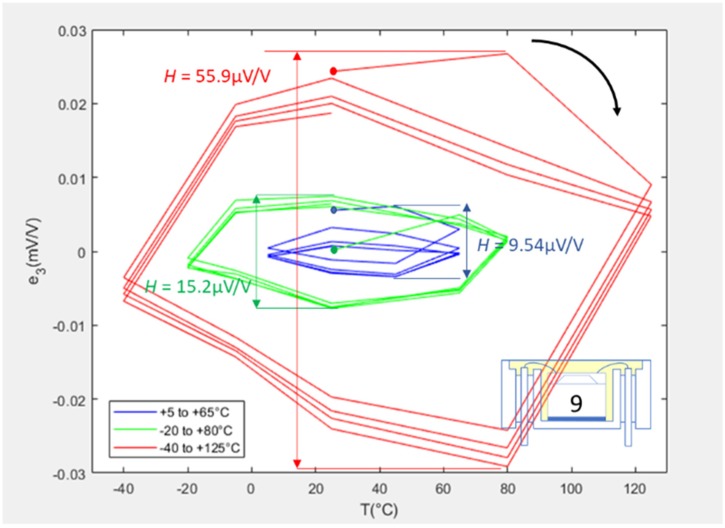
Error plots for SE #9 when L2 packaged.

Figure 8 shows error loops for the L2 packaged SE #9, for which the hysteresis values calculated for the three cycle ranges were 9.54, 15.2, and 55.9 μV/V. There was an initial phase of ratcheting of the loop for the LR cycle, which was attributed to the additional presence of the isolation membrane and the fluid fill, rather than the adhesive. Indeed, the adhesive was added in the L1 packaging stage and had already stabilised after the previous ER cycling. As the isolation membrane was welded onto the housing, the ratcheting was assumed to stem from the welded joint between the membrane and housing and not the fluid fill. By observing the decrease in the distance between consecutive points at 65 °C, it can be seen that it only took two cycles for these hysteresis loops to stabilise. As for the L1 package results, it was observed that the first points of the first cycle were significantly outside of the stabilised loops. It could only be concluded that a non-permanent physical phenomenon was occurring during that stage of the first cycle, which disappeared later in the cycling.

### 4.2. Evaluation of Packaging Stages on Consecutively Tested SEs

**Figure 9:**
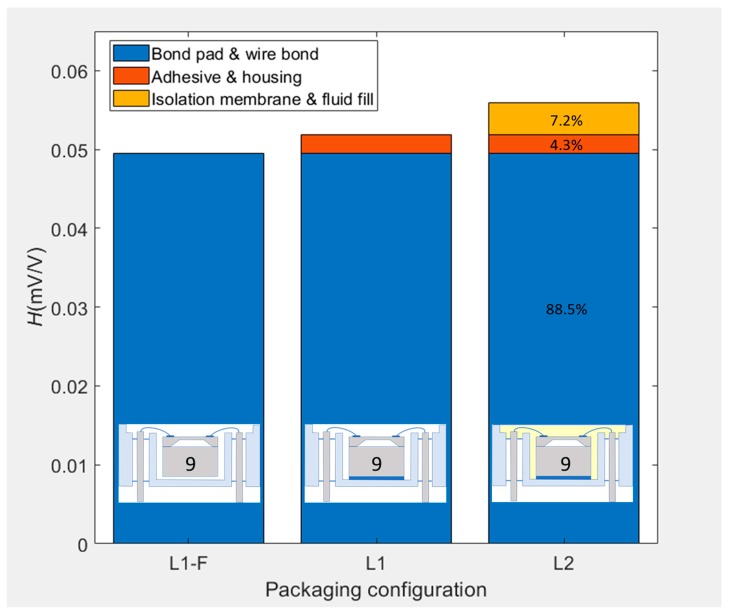
Stacked hysteresis values for −40 to 125 °C cycling of sensing element #9 for each packaging configuration.

Figure 9 shows the sequence of hysteresis values for SE #9 as it was consecutively packaged from L1-F to L2. Each bar represents the hysteresis value over the extended temperature range (i.e., the red loops of Figure 6, Figure 7 and Figure 8). Comparing packaging configurations to one another was deemed appropriate only for the cases where the loop exhibited a stabilised behaviour, which only occurred for the ER. The LR and IR were therefore not compared, as the ratcheting effect dominated the hysteresis value and one would be comparing the stabilisation of the packaging rather than actual hysteresis behaviour. Assuming the superposition principal holds true, it is possible to calculate the amount of hysteresis contributed by the individual groups of packaging elements. From a hysteresis value of 55.9 μV/V for the L2 package, 7.2% was attributed to the isolation membrane and the fluid fill, 4.3% was attributed to the adhesive and housing, and the remaining 88.5% was attributed to the bond pads and wire bonds. This result is of paramount importance, as it shows that further investigation of the L1-F packaging elements should be prioritised if hysteresis is to be significantly improved.

### 4.3. Thermal Stabilisation Due to Packaging Elements

**Figure 10:**
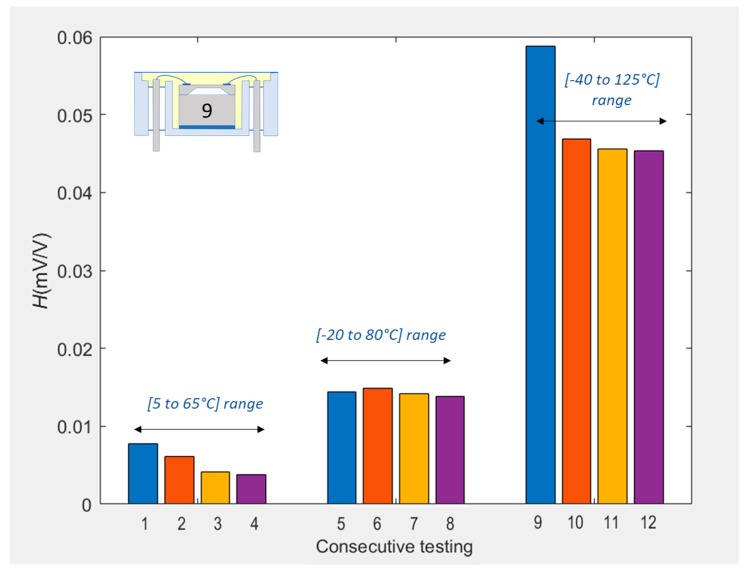
Hysteresis errors per cycle for the L2 packaged sensing element #9 sequentially subjected to the three temperature ranges. For each thermal range, the same colour of bar has been used for the same cycle number.

It was found that, when calculated for each individual cycle, hysteresis often decreased with repeated thermal cycling. Figure 10 shows the hysteresis value per cycle for SE #9 in the L2 configuration when consecutively cycled. It can be seen that, for the LR cycles, hysteresis reduced from 7.8 μV/V over the first cycle to 3.8 μV/V over the last; i.e., a total decrease of 51.3%. During the IR cycles, the unit exhibited a stable hysteresis of 14 μV/V, whereas for the ER cycles, hysteresis decreased from 58.7 μV/V over the first cycle to 45.4 μV/V over the last. This represented an overall reduction of 22.6%, most of which occurred between the first and second cycle.

## 5. Discussion

To the authors’ knowledge, this study is the first to consecutively package the same SEs to increasing levels in order to determine the relative contribution of the packaging elements to thermal hysteresis. Fitting is of paramount importance in the assessment of thermal hysteresis, as it enables the hysteresis to be confidently attributed to the packaging and not to uncertainties within the test set up. This good practice is not systematically applied, with some authors touching upon this matter [21,31] and others not [41]. To the authors’ knowledge, this study is also the first to do a systematic evaluation of increasing fitting orders on revealing thermal hysteresis, and to use the same fitting order on consecutively packaged sensors to compare the relative contributions of packaging elements. However, quantitative comparisons were judged to be acceptable only for instances in which the sensor exhibited stabilised, ratcheting-free thermal hysteresis loops.

### 5.1. Temperature and Humidity Effects on Hysteresis

Increased hysteresis for the wider temperature range cycles was expected, due to the increase in any package-related stress due to coefficient of thermal expansion (CTE) mismatches, which are then transmitted to the piezoresistors, thus changing the voltage output of the sensor. It is possible that the packaging materials are therefore subjected to viscoplastic and/or creep deformation, leading to history-dependant behaviour that is therefore very difficult to fit, as demonstrated by the pronounced hysteresis values. The ratcheting seen for the L1 package is thought to be due to the adhesive continuing to cure throughout both the LR and IR cycling, as the adhesive was initially cured at room temperature only. The ratcheting stopped after cycling the unit once over the ER, which suggests that one cycle at the extended range is more effective in stabilising the adhesive than multiple thermal cycles over narrower ranges. However, this could only be confirmed if the L1 configuration were to be cycled again in the LR and IR to observe whether the ratcheting effect had permanently disappeared. This test was unfortunately not undertaken prior to packaging the SEs into the L2 configuration. However, analysing the L2 package behaviour suggests that the repeatable loops seen for both the LR (after only the second cycle) and the IR, indicate that the adhesive had indeed stabilised after the ER in the L1 configuration. A suitable preconditioning step for the adhesive packaging element might therefore be to cycle it two or three times over the −40 to 125 °C range.

It should be noted that the L1 packages were exposed and therefore potentially sensitive to changes in humidity in the pressurising medium, as reported in the literature [42,43,44]. The testing apparatus applied relatively dry pressurised air, which could affect the level of moisture that may have accumulated within the adhesive during the rest time before the LR testing commenced. Therefore, the ratcheting effect cannot be conclusively attributed to temperature alone. It could be that the initial ratcheting effect is due to humidity being slowly desorbed from the adhesive when subjected to the dry air. As the initial humidity level of the adhesive was not controlled during this test, it would be necessary for future experiments to precondition the units such that testing of all exposed packages (L1-F and L1) would commence after stabilisation at the same humidity level.

### 5.2. Thermal Cycling Effects on Hysteresis

During the first cycle, the initial data points, as the temperature increased from 25 °C towards the maximum of the cycle, did not follow a similar line to the points recorded at the same temperatures in later cycles. This could be indicative of a relaxation phenomenon within the packaging elements, which would be expected to depend on the resting time between tests. Plastic deformation during the first cycle was ruled out, as the loops resumed a “stable” path after the second cycle onwards. It would be interesting to establish whether the deviation of the initial starting points is directly dependent on the resting time between tests and/or on the resting storage temperature. Similar tests could therefore be repeated after a period of resting time (e.g., three months) where the first cycle of the new test is compared to the last cycle of the previous test. This could indicate the presence of a non-permanent deformation behaviour (e.g., viscoelastic) of the packaging elements during storage. As a non-negligible part of the hysteresis is due to these initial data points, it could be argued that the calculation of the hysteresis accounting for all data points is too conservative. This could be conceded only if one was sure that all subsequent hysteresis loops, following a resting time period, were also stable.

The data indicates that the hysteresis observed in this study mainly stems from the elements present in the L1-F configuration, which are the Al–Si bond pads, the Al–Si wire bonds, and the borosilicate glass. As the borosilicate glass is CTE-matched to Si and is in contact with the underside of the SE away from the piezoresistors, this is not thought to contribute significantly to the hysteresis. However, due to the pronounced CTE mismatch (20.7 × 10^−6^ /°C) between the Al–Si and Si and the location of the bond pads on the active face of the SE and in close proximity to the piezoresistors, it is thought that the bond pads and/or the attached wire bonds are the main contributors to hysteresis.

The hysteresis could stem from the wire bonds exerting temperature-dependant stresses. Due to the CTE mismatch (8.5 × 10^−6^ /°C) between the housing and the Si, the effective distance between the bond pad and the feedthrough pin could change with temperature, leading to changes in the stresses transmitted from the wire bonds to the bond pad metallisations, and hence to the piezoresistors. This could be the case, especially for the L1-F configuration where the SEs were only supported by the wire bonds. It could be argued that an indirect way of assessing the effect of the wire bonds pulling on the L0 package would be to deliberately introduce changes in bond wire stresses.

Hysteresis could also stem from the bond pads experiencing stresses that are beyond their elastic limit [45,46,47]. Due to the manufacturing sequence of the L0 package, it is believed that returning to room temperature after both sintering the Al–Si and bonding to glass at elevated temperatures led to inelastic stress at least at the base of the bond pads where they are in contact with the Si. It therefore seems likely that the bond pads during the thermal cycling test are experiencing a combination of creep and plastic behaviour that could stem from dislocation alignment within the Al–Si bond pads as suggested by Khatibi et al. [48]. This behaviour would lead to the appearance of the hysteresis loops, the size of which would depend on the temperature points and the time spent at each point. Performing a cross section of the bond pad/Si interface may reveal any microstructural changes with electron microscopy, but was not possible in these experiments, as a destructive test such as this would impede the consecutive nature of the packaging methodology. However the existence of creep could be validated by conducting material analysis of the in-situ properties of the pad metallization, using, for example, nanoindentation at different temperatures and over time scales comparable to those applied during the thermal cycles. It would also be important to correlate any creep within the Al–Si to creep experiments for the SE in an L1-F packaging configuration. This would necessitate finite element modelling of at least the L1-F configuration, which should be capable of predicting creep in the Al–Si under different thermal conditions and the corresponding stresses transferred to the piezoresistors.

It could be argued that, because the temperature points in the IR and ER cycles for the L1-F package were not the same ones as for the L1 and L2 packages, the validity of the hysteresis values could be compromised. These results are worth analysing, as the finer granularity during the first cycle shows a rounded fitting error loop. Having more intermediate points, with each having a 1 h-dwell time, meant that the SEs experienced more time at both hotter and colder temperatures than for the L1 and L2 tests. This could mean that the hysteresis value is dependent on the time spent at each temperature, which would indicate that hysteresis is related to time dependent phenomena, such as creep, occurring predominantly within the bond pads and wires in the L1-F package.

### 5.3. Superposition Principal

The application of the superposition principal, as demonstrated by the bar graph in Figure 9, was thought to only be valid for cases where all packages reached a stabilised hysteresis loop, which was only observed for the ER cycles. It could be argued that if the L1-F package were to be inclined, certain wire bonds would support the weight of the SE more than others, leading to an asymmetric stress distribution. This effect would be prevented by adding the adhesive in the L1 package. It could therefore mean that the L1-F package is adding additional stresses that would not be present in the L1 and L2 packages, thereby invalidating the superposition principal; i.e., 88.5% of the 55.9 μV/V due to bond pad and wire bond effects may in reality reduce in magnitude, thereby increasing the contribution of the adhesive to a much higher percentage than 4.3%. This would be the case if the fitting function were to be unable to compensate for these orientation/inclination effects. It is for this reason that every packaged SE is fitted using a unique function with individualised fitting coefficients and always tested in the same orientation within the manifold. It would therefore be recommended to repeat the L1-F test cycles in different orientations and analyse the resulting hysteresis to see if the fitting function is able to accommodate these effects.

To prove the validity of the superposition principal, it would be interesting to rework the L2 packaged SEs back into L1 packages by removing the isolation membrane and releasing the fluid fill. If the hysteresis were found to return to that presented in Figure 7, it would further support the validity of the superposition principal. However, it is anticipated that the initial points may be slightly misaligned with the remaining loops, due to relaxation of package-related stresses.

## 6. Conclusions

To summarise the findings from this study, a novel packaging and testing methodology capable of revealing the cumulative effect of different groups of packaging elements on the hysteresis of a 50 to 200 kPa Si MEMS pressure sensor was demonstrated over three thermal cycling ranges. Five SEs were packaged consecutively at levels L1-F to L2 without being subjected to any previous thermal preconditioning. It was noted that the application of a mathematical fitting function is necessary in order to reveal the hysteresis behaviour and to compensate for testing set point uncertainties within the pressure and temperature sources. In this study, it was found that a “least squares” fitted third order polynomial function using the pressure, *P*, and diode voltage, *V_d_*, to allow extrapolation of the fitted output voltage, *Vf_out_*, was sufficient to reveal any fitting error, *e*, when compared to the sensor’s voltage output, *V_out_*. This error could then be used to identify any hysteresis, which was attributed directly to the effect of the packaging elements.

The effect on hysteresis of the individual packaging elements could not be entirely segregated for each packaging level. However, using a set of carefully selected assumptions regarding the combination of packaging elements, it was possible to pinpoint the effect of the adhesive contributing towards ratcheting and the bond pads and wire bonds as dominant contributors to the observed hysteresis. Assuming the validity of the superposition principle, 88.5% of the stabilised hysteresis of the complete L2 package at the extended temperature cycle range stemmed from the packaging elements of the initial L1-F configuration (i.e., the borosilicate glass, bond pad metallisation, and wire bonds). This was mainly thought to be driven by the high CTE mismatch between the SE and bond pad metallisation and the changing forces exerted by the wire bonds. Just 4.3% of the hysteresis was attributed to the additional packaging elements of the L1 configuration (i.e., the adhesive and the housing) and 7.2% to the packaging elements in the L2 configuration (i.e., fluid fill and isolation membrane). A reduction of thermal hysteresis was also witnessed due to repeated thermal cycling across all configurations and ranges.

## Figures and Tables

**Table 1:** Summary of packaging levels.

L0 Package	L1 Package
Schematic Diagram: 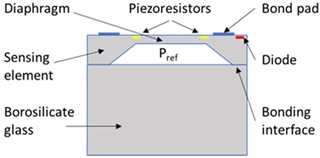	Schematic Diagram: 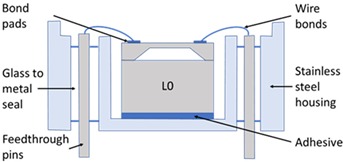
Packaging Elements: Al–Si bond pads + borosilicate glass	Packaging Elements: L0 + Al–Si wire bonds + adhesive + housing
***L2 Package***	***L1- Floating Package (L1-F)***
Schematic Diagram: 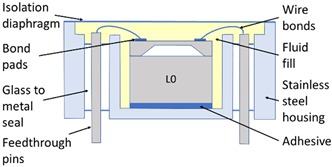	Schematic Diagram: 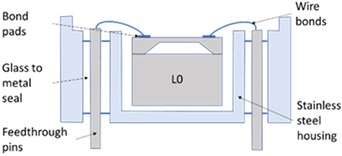
Packaging Elements: L1 + fluid fill + isolation diaphragm	Packaging Elements: L0 + Al–Si wire bonds

**Table 2:** Raw data collected at a nominal pressure of 100 kPa absolute for a typical L1-F sample when subjected to the first 5 to 65 °C cycle.

T (°C)	*P* (kPa)	*V_d_* (10^−1^V)	*V_S_* (V)	*V_b_* (10^−2^V)
25	100.0018	6.706131	4.316489	5.151264
45	100.0011	6.323966	4.356415	5.000526
65	100.0021	5.937537	4.405359	4.863641
45	100.0014	6.324579	4.356389	4.997359
25	100.0036	6.707893	4.316270	5.148594
5	100.0011	7.085253	4.287888	5.321121
25	100.0005	6.708597	4.316296	5.151588

## References

[1] KELLER Piezoresistive OEM Pressure Transducers, Series 7. http://www.keller-druck.com/picts/pdf/engl/3L_10L_e.pdf.

[2] NXP A Global Solution for Tire Pressure Monitoring Systems. https://www.nxp.com/docs/en/white-paper/TPMSWP.pdf.

[3] WIKA OEM Pressure Sensor with Ceramic Thick-film Technology Model SCT-1. https://www.wika.co.uk/sct_1_en_co.WIKA.

[4] TE CONNECTIVITY SENSORS MEAS M5600 Wireless Pressure Transducer. http://www.te.com/commerce/DocumentDelivery/DDEController?Action=showdoc&DocId=Data+Sheet%7FM5600%7FB8%7Fpdf%7FEnglish%7FENG_DS_M5600_B8.pdf%7FCAT-PTT0063.

[5] Wortman J.J., Evans R.A. (1965). Young’s modulus, Shear modulus, and Poisson’s ratio in Silicon and Germanium. J. Appl. Phys..

[6] Petersen K.E. (1982). Silicon as a Mechanical Material. Proc. IEEE.

[7] Hopcroft M.A., Nix W.D., Kenny T.W. (2010). What is the Young’s modulus of Silicon?. J. Microelectromech. Syst..

[8] Bridgman P.W. (1932). The Effect of Homogeneous Mechanical Stress on the Electrical Resistance of Crystals. Phys. Rev..

[9] Smith C.S. (1954). Piezoresistance Effect in Germanium and Silicon. Phys. Rev..

[10] Mason W.P., Thurston R.N. (1975). Use of Piezoresistive Materials in the Measurement of Displacement, Force, and Torque. J. Acoust. Soc. Am..

[11] Tufte O.N., Stelzer E.L. (1963). Piezoresistive Properties of Silicon Diffused Layers. J. Appl. Phys..

[12] Kanda Y. (1982). A Graphical Representation of the Piezoresistance Coefficients in Silicon. IEEE Trans. Electron Devices.

[13] Barlian A.A., Park W., Mallon J.R., Rastegar A.J., Pruitt B.L. (2009). Review: Semiconductor Piezoresistance for Microsystems. Proc. IEEE.

[14] Rowe A.C.H. (2014). Piezoresistance in silicon and its nanostructures. J. Mater. Res..

[15] Kumar S.S., Pant B.D. (2014). Design principles and considerations for the “ideal” silicon piezoresistive pressure sensor: A focused review. Microsyst. Technol..

[16] Kumar S.S., Pant B.D. (2014). Erratum to: Design principles and considerations for the “ideal” silicon piezoresistive pressure sensor: A focused review. Microsyst. Technol..

[17] Fraga M.A., Koberstein L.L. (2012). An overview on the Modeling of Silicon Piezoresistive Pressure Microsensors. Proceedings of the Workshop on Engineering Applications.

[18] Niu Z., Zhao Y., Tian B. (2014). Design optimization of high pressure and high temperature piezoresistive pressure sensor for high sensitivity. Rev. Sci. Instrum..

[19] Liu Y., Wang H., Zhao W., Qin H., Fang X. (2016). Thermal-Performance Instability in Piezoresistive Sensors: Inducement and Improvement. Sensors.

[20] Krondorfer R.H., Kim Y.K. (2007). Packaging Effect on MEMS Pressure Sensor Performance. IEEE Trans. Compon. Packag. Technol..

[21] Sandvand Å., Halvorsen E., Jakobsen H. (2017). In Situ Observation of Metal Properties in a Piezoresistive Pressure Sensor. J. Microelectromechanical Syst..

[22] Lishchynska M., O’Mahony C., Slattery O., Wittler O., Walter H. (2007). Evaluation of Packaging Effect on MEMS Performance: Simulation and Experimental Study. IEEE Trans. Adv. Packag..

[23] Sandvand Å., Halvorsen E., Aasmundtveit K.E., Jakobsen H. (2015). Influence of Glass-Frit Material Distribution on the Performance of Precision Piezoresistive MEMS Pressure Sensors. IEEE Trans. Compon. Packag. Manuf. Technol..

[24] Chou T.-L., Chu C.-H., Lin C.-T., Chiang K.-N. (2009). Sensitivity analysis of packaging effect of silicon-based piezoresistive pressure sensor. Sensors Actuators A Phys..

[25] Li B., Zhang G.Q., Yang D.G., Hou F., Hai Y. (2010). The effect of Diaphragm on Performance of MEMS Pressure Sensor Packaging. Proceedings of the 2010 11th International Conference on Electronic Packaging Technology & High Density Packaging.

[26] Zhang Z., Liu C., Wan Z., Cao G., Lu Y., Song B., Liu S. (2010). Optimization of Packaging Process of Piezoresistive Engine Oil Pressure Sensor. Proceedings of the 2010 11th International Conference on Electronic Packaging Technology & High Density Packaging.

[27] Hamid Y., Hutt D.A., Whalley D.C., Craddock R. Effect of Thin Film Interconnect Inelasticity on MEMS Pressure Sensor Hysteresis. Proceedings of the 5th International Conference on Sensorsand Electronic Instrumentation Advances.

[28] Schroder S., Niklaus F., Nafari A., Westby E.R., Fischer A.C., Stemme G., Haasl S. (2015). Stress-Minimized Packaging of Inertial Sensors by Double-Sided Bond Wire Attachment. J. Microelectromech. Syst..

[29] Lee C.-C., Peng C.-T., Chiang K.-N. (2006). Packaging effect investigation of CMOS compatible pressure sensor using flip chip and flex circuit board technologies. Sensors Actuators A Phys..

[30] Rohwer L.E.S., Chu D. (2011). Thin Gold to Gold Bonding for Flip Chip Applications. Proceedings of the 2011 IEEE 61st Electronic Components and Technology Conference (ECTC).

[31] Waber T., Pahl W., Schmidt M., Feiertag G., Stufler S., Dudek R., Leidl A. (2014). Temperature characterization of flip-chip packaged piezoresistive barometric pressure sensors. Microsyst. Technol..

[32] Bourgeois C., Hermann J., Blanc N., De Rooij N.F., Rudolf F. (1995). Determination of The Elastic Temperature Coefficients of Monocrystalline Silicon. Proceedings of the 8th International Conference Solid-State Sensors Actuators, Eurosensors IX.

[33] KULITE Pressure Transducer Handbook. https://www.kulite.com/document_category/kulite-pressure-transducer-handbook.

[34] Chiang H.-N., Chou T.-L., Lin C.-T., Chiang K.-N. (2008). Investigation of the Hysteresis Phenomenon of a Silicon-based Piezoresistive Pressure Sensor. Proceedings of the International Microsystems, Packaging, Assembly and Circuits Technology Conference.

[35] Song J.W., Lee J.-S., An J.-E., Park C.G. (2015). Design of a MEMS piezoresistive differential pressure sensor with small thermal hysteresis for air data modules. Rev. Sci. Instrum..

[36] General Electric UNIK 5000 Pressure Sensing Platform. https://www.industrial.ai/sites/g/files/cozyhq596/files/acquiadam_assets/unik_5000_datasheet.pdf.

[37] KELLER Highly Precise Pressure Transmitters Series 33 X/35 X. http://www.keller-druck.com/picts/pdf/engl/33xe.pdf.

[38] Chiou J.A., Chen S. (2008). Pressure nonlinearity of micromachined piezoresistive pressure sensors with thin diaphragms under high residual stresses. Sensors Actuators A Phys..

[39] Hamid Y., Hutt D.A., Whalley D.C., Craddock R. Packaging Effects on MEMS Pressure Sensor Hysteresis. Proceedings of the 2019 IEEE 21st Electronics Packaging Technology Conference (EPTC).

[40] Sandvand Å., Halvorsen E., Aasmundtveit K.E., Jakobsen H. (2017). Identification and Elimination of Hygro-Thermo-Mechanical Stress-Effects in a High-Precision MEMS Pressure Sensor. J. Microelectromech. Syst..

[41] Chiou J.A., Chen S. (2005). Thermal Hysteresis Analysis of MEMS Pressure Sensors. J. Microelectromech. Syst..

[42] Mubashar A., Ashcroft I.A., Critchlow G.W., Crocombe A.D. (2009). Moisture absorption-desorption effects in adhesive joints. Int. J. Adhes. Adhes..

[43] Keller J., Mrossko R., Dobrinski H., Stürmann J., Döring R., Dudek R., Rzepka S., Michel B. (2013). Effect of moisture swelling on MEMS packaging and integrated sensors. Microelectron. Reliab..

[44] Chao L., Lin C., Lau Y. (2005). A Study on the Effects of Humidity, Temperature, and Pressure Sensor on the Piezoresistive Film Co-Structure. Proceedings of the 2005 International Conference on MEMS, NANO and Smart Systems.

[45] Gardner D.S., Flinn P.A. (1988). Mechanical Stress as a Function of Temperature in Aluminum Films. IEEE Trans. Electron Devices.

[46] Bader S., Kalaugher E.M., Arzt E. (1995). Comparison of mechanical properties and microstructure of Al(1 wt.%Si) and Al(1 wt.%Si, 0.5 wt.%Cu) thin films. Thin Solid Films.

[47] Eiper E., Resel R., Eisenmenger-Sittner C., Hafok M., Keckes J. (2004). Thermally-induced stresses in thin aluminum layers grown on Silicon. Powder Diffr..

[48] Khatibi G., Weiss B., Bernardi J., Schwarz S. (2012). Microstructural Investigation of Interfacial Features in Al Wire Bonds. J. Electron. Mater..

